# The Search for Consistency in Residual Symptoms in Major Depressive Disorder: A Narrative Review

**DOI:** 10.3390/jpm14080828

**Published:** 2024-08-04

**Authors:** Michał Pastuszak, Wiesław Jerzy Cubała, Aleksander Kwaśny, Agnieszka Mechlińska

**Affiliations:** Department of Psychiatry, Faculty of Medicine, Medical University of Gdańsk, 80-214 Gdańsk, Poland; michalpastuszak@gumed.edu.pl (M.P.); cubala@gumed.edu.pl (W.J.C.); agnieszka.mechlinska@gumed.edu.pl (A.M.)

**Keywords:** residual symptoms, depression, major depressive disorder, predictor, functional impairment, recovery, relapse

## Abstract

Residual symptoms are prevalent in major depressive disorder (MDD), encompassing a wide spectrum of symptoms such as sleep disturbances, changes in weight and appetite, cognitive impairment, and anxiety. These symptoms consistently impair daily functioning, diminish quality of life, and forecast disease relapse. Despite their clinical significance, residual symptoms lack a unified definition, potentially leading to confusion with treatment-emergent symptoms and ambiguity across studies, thereby hindering the generalizability of research findings. While some research identifies insomnia and mood disturbances as critical indicators, other studies emphasize different symptoms or find no significant correlation. Inconsistencies in defining residual symptoms, as well as methodological differences across studies, contribute to these conflicting results. While clinicians focus on alleviating negative symptoms to improve functional status, patients often prioritize achieving positive affect and overall well-being as essential components of successful treatment. It necessitates a comprehensive approach to patient care in depression. This review explores the phenomenon of residual symptoms in MDD, focusing on the ambiguity in definitions, clinical characteristics, and their impact on long-term outcomes. The lack of a standardized regulatory or academic definition for residual symptoms leads to varied interpretations among clinicians, underscoring the need for standardized terminology to guide effective treatment strategies and future research.

## 1. Introduction

Neuropsychiatric conditions impose the most substantial global burden of disease. Prominently, major depressive disorder (MDD) affects nearly 300 million individuals worldwide [1]. Annually, up to 60% of patients with MDD may experience work-related impairments, leading to an average loss of over four workdays per week due to the disease [2]. The public health implications stem from the well-established link between MDD and several common chronic physical diseases [3,4]. For instance, MDD is a known risk factor for cardiovascular disease, obesity, and type 2 diabetes mellitus, particularly in individuals with more severe or persistent depressive syndromes [5].

In the Sequenced Treatment Alternatives to Relieve Depression (STAR*D) trial, which primarily utilized traditionally acting antidepressants (TAAD), remission rates for the first, second, third, and fourth acute treatment steps were 36.8%, 30.6%, 13.7%, and 13.0%, respectively. The overall cumulative remission rate was 67%. Patients who required multiple treatment steps experienced higher relapse rates during the naturalistic follow-up phase [6]. Notably, 90% of participants who reached remission and response status experienced at least one residual symptom with appetite/weight disturbance, sad mood, decreased energy, and decreased concentration among the most common residual symptoms [7,8].

A significant number of patients experience treatment-resistant depression (TRD), defined as the failure to respond to two adequate pharmacological treatments [9]. Its prevalence varies due to differing definitions [10] but remains substantial globally, contributing significantly to the economic burden of MDD due to higher indirect costs and greater psychosocial impairment [11]. For instance, nearly 3 million adult patients suffer from TRD in the United States, which contributes to almost USD 44 billion annually [12]. Consequently, there has been increased interest in recent advancements, including rapid-acting antidepressants (RAAD) such as ketamine, noted for its antidepressive, antisuicidal, and antianhedonic effects [10,13,14], as well as a burgeoning interest in psychedelics as prospective therapeutic agents [15], particularly for patients who do not respond to TAAD.

Regardless of the severity, chronicity, or stage of depression, it is imperative that treatment focuses on promoting functional recovery, emphasizing the restoration of everyday activities and overall well-being [16]. Despite positive responses to pharmacological treatment and even formal remission in cases of TRD, residual symptoms persist in nearly all patients [7,8,16]. These persistent symptoms significantly burden patients, impacting their quality of life and daily functioning. Consequently, there is a crucial need for continued treatment optimization to achieve full symptom resolution and functional recovery, ensuring that patients can be free from the lingering effects of depression.

However, the area of residual symptoms remains under-researched. Current reports are relatively scarce, and the existing literature is not well-established in terms of defining residual symptoms, their incidence, and how they impact patients, often presenting conflicting results. This review aims to explore the phenomenon of residual symptoms, with a specific focus on the ambiguity in definitions, clinical characteristics, and their impact on long-term outcomes.

## 2. Methodology

Electronic databases, PubMed and Web of Science, were searched from their inception until June 2024. Only English-language papers were considered. There were no restrictions on the publication date. The following keywords were used in various combinations: residual symptoms, relapse, recurrence, functional impairment, depression, major depression, and major depressive disorder. Inclusion criteria encompassed (1) adult subjects, (2) MDD diagnosis with residual symptoms, and (3) data on incidence or functional impairment or relapse/recurrence prediction. Exclusion criteria included: (1) patients below 18 years of age, (2) diagnosis other than MDD, (3) lack of residual symptoms. Titles and abstracts of relevant papers were subsequently screened, with the most prominent papers included in the review.

## 3. Residual Symptoms 

### 3.1. Inconsistency in Definitions

Characterizing change during the treatment of depression is defined diversely. For example, formal remission is characterized by cut-off scores in standardized scales (e.g., ≤10 points in Montgomery–Åsberg Depression Rating Scale (MADRS)), and treatment response is defined as a reduction of at least 50% from the baseline score or partial response, typically defined as an improvement between 25 and 50% [17].

Nevertheless, the identification of residual symptoms is inconsistent across the literature. Examples of these definitions are summarized in Table 1. For instance, patients were considered to exhibit residual symptoms if they met the criteria for formal remission on the Beck Depression Inventory (BDI) [18] the 17-item Hamilton Depression Rating Scale (HAM-D) [19,20,21], the 16-item Quick Inventory of Depressive Symptomatology-Self Report (QIDS-SR16) [8,22], 16-item Quick Inventory of Depressive Symptomatology-Clinician (QIDS-C16) or Harvard Department of Psychiatry/National Depression Screening Day (HANDS) [23]. Another common method for classifying residual symptoms is based on treatment response in the QIDS-SR16 [7,24], MADRS [25], HAM-D [26], or the Visual Analogue Scale (VAS) [27,28]. The next definition includes partial response; however, some studies do not explicitly state what definition they followed [29,30]. Additionally, there are studies that do not fit into any specific category. For instance, two studies used the Psychiatric Status Rating (PSR) scale with different thresholds [16,31]. Another study defined partial remission as subjectively reported improvement in symptoms over at least 2 months with a BDI score of less than 20 [32]. Other studies employed different cut-off scores for residual symptoms on the HAM-D than the score required for formal remission [33] or did not explicitly describe criteria for the presence of residual symptoms in the non-remitters group, i.e., it is not clear if this group consisted of responders only or all patients without formal remission [34]. 

### 3.2. Clinical Characteristics

#### 3.2.1. Patient-Reported Outcomes

In the aforementioned STAR*D study, among patients who achieved remission after 12 weeks of treatment with citalopram, less than 10% reported complete resolution of depressive symptoms in QIDS-SR16. The most frequent complaints among the remaining remitters included weight gain (71.3%), middle insomnia (54.9%), increased appetite (50.6%), difficulty falling asleep (29.5%), and persistent sad mood (27.1%) [8]. Conversely, among patients who responded to treatment but did not achieve remission, the most frequently persistent depressive symptoms were mid-nocturnal insomnia (81.6%), sad mood (70.8%), and decreased concentration/decision-making (70.6%) [7].

In a study conducted by Xiao et al. [27], individuals who achieved remission, as measured by the Visual Analogue Scale (VAS), commonly experienced residual symptoms in the QIDS-SR16 with at least minimal intensity, including middle insomnia (39.4%), early insomnia (32.8%), decreased energy (32.3%), and decreased concentration (31.3%). Conversely, among non-remitters (i.e., patients who responded by at least 50% but did not achieve remission), the most prevalent residual symptoms were decreased concentration (82.4%) and decreased energy (79.6%). Additionally, among the 15 somatic symptoms assessed using the Patient Health Questionnaire (PHQ), individuals achieving remission most commonly reported feeling tired (35.5%), trouble sleeping (32.6%), headache (31.9%), intestinal problems (31.3%), palpitations (26.3%), gastric discomfort (22.3%), dizziness (22.2%), and stomach ache (20.6%). Similar results were observed for non-remitters [27]. Another study found that “concentration/decision making” was the most prominent and thus the core residual symptom [22]. In another study, the most frequently reported residual symptoms included sleep disorders, depressed mood, biological symptoms, inattention, poor self-esteem, loss of interest, decreased energy, and mental anxiety. These symptoms were reported with higher frequency in patients with functional impairment [28].

#### 3.2.2. Clinician-Rated Outcomes

In a randomized controlled trial (RCT) involving fluoxetine, over 90% of patients in remission had at least one residual depressive symptom, with the most common being sleep disturbances (both insomnia and hypersomnia) and anxiety [19]. Another RCT aimed at verifying the effectiveness of gabapentin and clonazepam for residual sleep disturbances found that patients suffered from significant sleep issues at baseline, as measured by the Pittsburgh Sleep Quality Index (PSQI) and the Insomnia Severity Index (ISI) [33]. In a study that included MDD and/or dysthymic disorders patients, the most common symptom domains were core mood symptoms, insomnia symptoms, anxiety symptoms, and somatic symptoms [34]. Similarly, Romera et al. [26] reported that the most frequent residual symptom was anxiety, followed by core mood symptoms, residual insomnia, and somatic symptoms.

#### 3.2.3. Patient-Reported and Clinician-Rated Outcomes

There are discrepancies between clinicians’ and patients’ perspectives, with patients reporting symptoms as more severe than clinicians do [35], therefore it is pragmatic to combine both perspectives. For example, residual symptoms among patients diagnosed with MDD who underwent repetitive transcranial magnetic stimulation (rTMS) were evaluated using both the QIDS-SR16 and the 28-item Hamilton Depression Rating Scale (HAM-D28). The most commonly reported residual symptoms in the QIDS-SR16 included mid-nocturnal insomnia (70.6%), sad mood (64.7%), decreased concentration/decision-making (61.8%), and low energy (51.5%). Similarly, the most frequently reported residual symptoms in the HAM-D28 were depressed mood (61.8%), diurnal variation (54.4%), and feelings of guilt (50.0%) [21].

**Figure 1:**
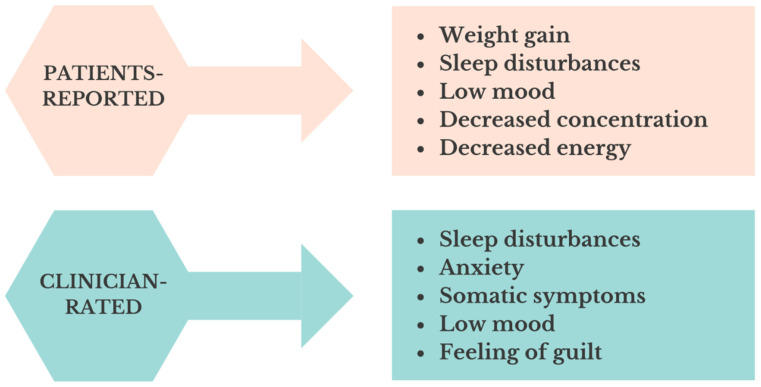
The diagram depicts the prevalence of the five most frequently reported residual symptoms from both patients’ and clinicians’ perspectives across standardized depression rating scales.

Although there is a greater volume of data on self-reported residual symptoms compared to clinician-rated outcomes, likely due to practical reasons related to data collection, there appears to be a notable overlap in the reporting of residual symptoms from both perspectives. A summary of the most consistently and commonly reported symptoms is presented in Figure 1.

### 3.3. Functional Impairment

Residual symptoms contribute greatly to impairment in professional and leisure time activities. For example, non-remitters exhibited notably higher levels of functional impairment compared to remitters across general functioning and all three functional domains in the Sheehan Disability Scale (SDS), i.e., work, social life, and family life. Non-remitters experienced more frequent occurrences of days lost due to illness and underproductivity in both general functioning and across all three domains [27]. The factors most closely related to functional impairment included loss of interest, anxiety, and sleep disorders [28]. Patients with non-remitted MDD, particularly those experiencing more severe residual somatic symptoms, exhibit pronounced impairments in quality of life and increased clinical symptomatology [36].

A single study explored the correlation between specific residual symptoms and patient functioning. This study identified a stronger association with residual core mood symptoms. Residual insomnia showed a weaker relationship with patient functioning, while residual somatic symptoms were not associated. Importantly, residual insomnia was found to have a significantly weaker association with patient functioning compared to residual core mood symptoms. The absence of pain significantly increased the likelihood of normal functioning, regardless of residual anxiety. However, the absence of residual anxiety improved the chances of normal functioning only in the absence of pain. The link between residual symptoms and particular domains of functional impairment was not studied [26].

### 3.4. Predictors of Relapse and Recurrence

Residual symptoms in psychiatric disorders like depression signal ongoing illness activity and suggest a heightened risk of relapse, making them crucial for identifying patients who are more likely to experience a recurrence. Consistent findings in the literature suggest that the presence of residual symptoms leads to a quicker relapse of depressive episodes, highlighting their role in the active phase of the illness. Moreover, the characteristics and number of residual symptom domains further influence the likelihood of relapse. Research indicates that individuals with a broader range of lingering symptoms following initial treatment are at a higher risk of experiencing a relapse [8]. The impact of these residual symptoms on relapse risk appears consistent across different methods of assessment, whether self-reported or clinician-rated [37]. For instance, research shows that sleep disturbances and feelings of lassitude significantly increase the likelihood of relapse following electroconvulsive therapy in older adults with depression [25]. Similarly, residual symptoms such as restlessness, insomnia, and changes in weight are recognized as markers that can better pinpoint individuals with MDD susceptible to relapse [37]. Studies have highlighted insomnia specifically as a significant predictor of recurrence in individuals recovering from recurrent major depression [20]. Furthermore, broader aspects of the depressive mood spectrum, including residual obsessive-compulsive and phobic anxiety symptoms, also contribute to an elevated risk of relapse [38,39,40]. In contrast, patients who achieve asymptomatic recovery tend to experience a longer period before any recurrence of depressive episodes occurs [18,31].

However, there are areas of controversy in the literature regarding whether the total number of residual symptoms predicts relapse or recurrence universally. Some studies suggest no significant predictive value for overall symptom burden [19,20], indicating variability in how residual symptoms may impact the course of depression. In addition, there are contradictory findings regarding the predictive value of residual mood and anxiety symptoms across different studies [20], as well as inconsistencies in the role of sleep disturbances in predicting relapse [8,19]

The field of relapse prediction is characterized by inconsistent findings. Supportive data is frequently contradicted by subsequent studies. It is undoubted that residual symptoms signal an increased risk of relapse in depressive episodes, and their identification and overall clinical characterization are necessary. However, there is controversy regarding the designation of a single symptom or symptom group as definitive indicators of relapse.

### 3.5. Discontinuation Syndrome and Residual Symptoms 

The distinction between treatment-emergent symptoms, discontinuation symptoms, and residual symptomatology remains ambiguous. Practitioners often consider many of these symptoms as residual. The primary challenge lies in differentiating overlapping complaints such as emotional blunting and anhedonia, since both conditions share phenotypic similarities. However, anhedonia is a core symptom of depression, whereas emotional blunting is a side effect of selective serotonin reuptake inhibitors (SSRIs) [41]. Despite the complexities in distinguishing these symptoms, both treatment-emergent and discontinuation symptoms significantly impact patients’ quality of life [42]. To achieve this, it is essential to employ systematic and quantitative monitoring of depressive symptoms using standardized assessment tools. These tools help to provide a clear and objective measure of symptom presence and severity, facilitating better differentiation between symptom types. One such tool is the Discontinuation-Emergent Signs and Symptoms (DESS) inventory, which is particularly beneficial in routine psychiatric practice. The DESS inventory is designed to specifically assess symptoms that emerge during SSRI discontinuation, thereby aiding in the effective evaluation of the effects of drug tapering. Its use allows clinicians to distinguish between discontinuation symptoms and residual depressive symptoms more accurately [43].

### 3.6. Successful Outcome Measure

With the advancement of pharmacological treatments for depression, the measurement of treatment success has evolved from simply assessing response to aiming for remission and ultimately achieving full functional recovery [44,45]. Response is defined as a significant reduction in depressive symptoms, typically a 50% decrease from baseline on a standardized rating scale. It offers a quantifiable measure of short-term improvement and guides treatment adjustments; however, it may not capture the full resolution of symptoms or the impact on quality of life. Remission is defined as a period where depressive symptoms fall below a specific threshold, signaling substantial symptom reduction and improved overall functioning; however, it may still leave residual symptoms affecting daily life and suffers from variability in criteria across studies and settings. Full functional recovery involves achieving both symptom remission and the restoration of normal functioning in personal, social, and occupational domains. It offers a comprehensive measure of treatment success by focusing on overall quality of life but is more challenging to measure and requires alignment with individual patient goals and expectations. According to Canadian Network for Mood and Anxiety Treatment (CANMAT) guidelines, full remission should be considered both in terms of short-term and long-term effects, as short-term remission must lead to sustained full recovery in the long term [46]. The best chance for full recovery from a depressive episode occurs within the first 3–6 months. After one year, the likelihood of full recovery decreases to approximately 10–15%, and after two years, the likelihood of full recovery drops to a single-digit percentage [47]. Therefore, the earlier treatment is initiated, the better the prospects for future outcomes. However, healthcare professionals and patients have differing expectations regarding the definition of remission and recovery. Physicians aim to alleviate and minimize depressive symptoms, considering formal remission with residual symptoms as a promising outcome. However, patients prioritize positive affect and have distinct expectations compared to physicians [48]. Therefore, not only the symptom-centered approach, but patients’ values, needs, and preferences should be considered when making treatment decisions and assessing outcomes to ensure patient-centered and individualized care [49].

## 4. Discussion

This review provides a comprehensive overview of the literature on residual symptoms in MDD. Residual symptoms lack a standardized definition and are defined diversely by clinicians. Sleep disturbances, changes in weight and appetite, cognitive impairment, low mood, anxiety, decreased energy, and somatic symptoms consistently emerge as the most common residual symptoms, regardless of whether they are reported by patients or assessed by clinicians. Residual symptoms such as sleep disturbances, cognitive impairments, and mood disturbances have a profound impact on individuals’ daily functioning and overall quality of life. They often result in reduced productivity at work or school due to difficulty concentrating, making decisions, or maintaining motivation. Moreover, these symptoms frequently lead to increased absenteeism from work.

The field of relapse prediction is characterized by inconsistent findings. Interestingly, the symptoms contributing to this risk vary between studies. This variability can result in differing conclusions about which residual symptoms are most crucial for predicting relapse. The lack of a well-grounded and commonly used definition is a confounding factor. Since some studies use formal remission as a threshold for the presence of residual symptoms [8,21], others use the response as a criterium [7,25], and some studies use yet another criterium [32], it must be acknowledged as a confounding factor in the research of residual symptoms. The complexity increases when considering that remission and response outcomes are defined from multiple perspectives—clinician versus patient—and assessed using various tools that may not encompass the same aspects of the illness. Clinicians and patients may have differing views on what constitutes remission or response, leading to potential discrepancies in treatment evaluations. Furthermore, assessment tools used by clinicians may focus on specific symptom domains or severity thresholds that do not align with those considered by patients, resulting in incomplete or inconsistent coverage of the disease spectrum. This divergence complicates the accurate assessment of treatment outcomes and the development of tailored treatment strategies.

Residual symptoms are evaluated through both self-report measures [7,8] and clinical assessments [25], which may result in differing evaluations of the final outcome. From a clinical perspective, patients in formal remission and those who respond to treatment might differ significantly, presenting distinct spectra and intensities of depressive symptoms. This suggests that residual symptoms in remitters and responders could represent two clinically distinct phenomena, with responders exhibiting somewhat different but still active forms of the disease. Patients who continue to experience residual depressive symptoms often exhibit a higher disease burden and increased functional impairment compared to those who have achieved full remission. Remitters, by definition, do not meet the criteria for a depressive episode and typically experience fewer symptoms and less impairment. However, comparing these two groups can be challenging due to the differences in symptom profiles, severity, and impact on daily functioning. Consequently, the assessment and management of residual symptoms in patients with ongoing depressive symptoms must be approached with caution, recognizing the complexities and variabilities inherent in these conditions. Potentially, patients who respond to treatment but continue to exhibit residual symptoms may require management similar to that of patients with active depression. In contrast, remitters with residual symptoms, who present with a more limited symptom profile, need a distinctly individualized treatment approach. Furthermore, with the advent of rapid-acting antidepressants (RAADs) and the increasing interest in psychedelic treatments, the standard criteria for residual symptoms may not always be applicable. For example, when patients show significant improvement within hours after receiving ketamine or 5-methoxy-N,N-dimethyltryptamine (5-MeO-DMT) [10,50], it is debatable whether symptoms that persist shortly after such rapid improvement should be classified as residual. However, there has not yet been an established timeframe for the onset of residual symptoms. It could be argued that a standardized definition of residual symptoms should include a specific cut-off score on depression scales that preferably reflects formal remission and covers a particular timeframe, making it applicable for use in RAAD research.

**Figure 2:**
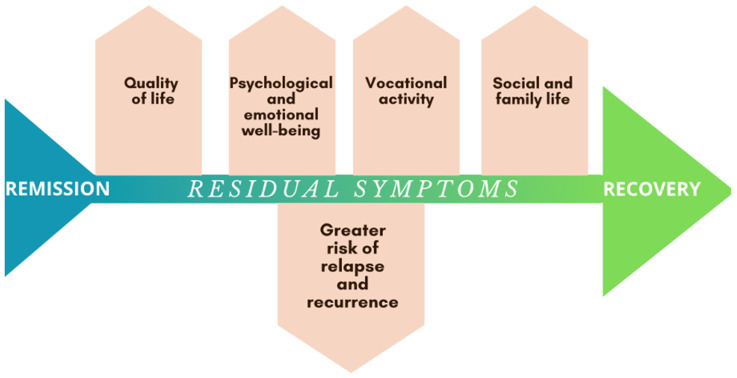
The diagram illustrates how residual symptoms influence various domains of life. Despite achieving remission, the presence of residual symptoms can increase the risk of relapse and recurrence, impeding full recovery and negatively affecting overall well-being.

Among all depressive symptoms, sleep disturbances are the most consistently reported residual symptom [7,8] with mid-nocturnal insomnia being the most common presentation [51]. While statistics vary, anxiety, low mood, cognitive impairment, and weight changes are also frequently reported in the literature. However, few studies distinguish and report the prevalence of treatment-emergent symptoms [7,8]. This distinction is essential because, as McClintock et al. [7] report, insomnia is both the most common residual symptom and a common treatment-emergent symptom. If treatment-emergent symptoms are not analyzed separately from residual symptoms, the incidence of some residual symptoms may be inaccurately elevated. Patients with TRD experience a high disease burden, low health-related quality of life, and reduced functioning and productivity, with a significant proportion being unable to work [2]. In this context, patients with residual symptoms exhibit similarities to those with TRD, as both groups experience significant functional impairment and a lowered quality of life [27,36]. Given that residual symptoms are widely recognized as predictors of relapse and recurrence, it is prominent that the primary goal of treatment should be functional recovery. Research indicates that functional recovery is achievable even in patients with TRD [16,18]. This underscores the importance of addressing residual symptoms comprehensively to improve long-term outcomes and overall quality of life in depressive disorders as depicted in Figure 2.

Clinicians typically measure the success of depression treatment by achieving remission and recovery, which they define primarily as the reduction or elimination of depressive symptoms. This clinical perspective focuses on quantifiable changes in symptom severity, using standardized scales and assessment tools to determine whether a patient no longer meets the criteria for depression. The ultimate goal from the clinician’s viewpoint is to alleviate the negative symptoms that define the disorder, thereby improving the patient’s functional status and overall quality of life. However, patients often have a different perspective on what constitutes successful treatment. While the absence of depressive symptoms is undoubtedly important, many patients place a higher value on the presence of positive affect—experiencing joy, interest, and engagement with life. For them, functional recovery is not just about reducing or eliminating negative symptoms but also about regaining a sense of well-being and the ability to enjoy life fully. This emphasis on positive affect highlights a more holistic view of recovery, one that encompasses both the removal of distress and the promotion of positive emotional experiences.

This review has its limitations. Firstly, electronic databases were not searched systematically, potentially omitting relevant studies that could have added value. Secondly, the domain of residual symptoms is highly inconsistent, complicating the ability to draw definitive conclusions. Nevertheless, the review has strengths, including the identification of discrepancies in definitions, an overview of the incidence and impact of residual symptoms on patient functioning and relapse risk, and a contextualization of these symptoms in light of recent advancements in RAADs. Further research should focus on establishing a common regulatory and/or academic definition for residual symptoms to improve the quality and clarity of research in this area. This would allow for a unification of findings and provide more comprehensive outcomes in the field. Only by ensuring we use consistent terminology can we develop and implement appropriate treatment strategies. Otherwise, the range of treatment options may remain as diverse and under-researched as seen in residual insomnia [51]. Additionally, since residual symptoms are typically of mild severity, it is important to develop a tool that can accurately capture these symptoms and their impact on functioning, even at low levels. Developing a sensitive and comprehensive tool to capture residual symptoms mild in severity and their impact on functioning will provide a more nuanced understanding and management of the patient’s condition.

## 5. Conclusions

Common residual symptoms such as sleep disturbances, changes in weight and appetite, cognitive impairments, low mood, anxiety, decreased energy, and somatic complaints consistently appear in both patient reports and clinician assessments, significantly impacting daily functioning and overall quality of life. The field of relapse prediction is marked by inconsistent findings, with the most robust evidence relating to sleep disturbances. Despite the conflicting literature, substantial evidence suggests that residual symptoms, especially when numerous, are strong predictors of relapse and recurrence in depressive episodes. However, the lack of a standardized definition for residual symptoms leads to varied interpretations among clinicians, and the predictive value of specific symptoms remains controversial. It can be assumed that overall symptom burden is a more significant factor, and only by standardizing terminology can we develop and implement effective treatment strategies.

## Figures and Tables

**Table 1:** Definitions of residual symptoms used in the literature.

Study	Presence of Residual Symptoms Definition
Paykel et al., 1995 [18]	Formal remission in BDI
Judd et al., 1998 [31]	Score of 2 on PSR Scale
DeBattista et al., 2003 [29]	Partial response *
Fava et al., 2005 [30]	Partial response *
Fava et al., 2006 [23]	Partial or full remission (both considered as response to treatment) Response defined as score < 9 on HANDS
Dombrovski et al., 2008 [20]	Remission defined as HDRS-17 score ≤ 7
Iovieno et al., 2010 [19]	Formal remission in HAM-D17 (score < 7)
Nierenberg et al., 2010 [8]	Formal remission defined as a QIDS-SR16 score of ≤5
McClintock et al., 2011 [7]	Treatment response defined as a 50% or greater reduction in the baseline QIDS-SR16
Fekadu et al., 2011 [16]	Subthreshold PSR score of 3 to 4
Britton et al., 2012 [32]	Partial remission was defined by a subjectively reported improvement in symptoms in the last 2 months, BDI score ≤ 20 and the exclusion of individuals with severely depressed mood/anhedonia, or active suicidal ideation
Romera et al., 2013 [26]	Improvement above 50% in HAM-D
Mowla et al., 2015 [33]	Score in HDRS < 10
Hiranyatheb et al., 2016 [34]	Remission defined as HDRS-17 score ≤ 7 Criteria for non-remitters group not stated
Sakurai et al., 2022 [21]	Remission was defined as a QIDS-C16 total score of ≤5
Xiao et al., 2018 [27]	Patients who responded to antidepressant drug treatment reporting improvement of depressive symptoms of ≥50% on the VAS were divided into ‘remitters’ (QIDS-SR total score of ≤5) and ‘non-remitters’ (QIDS-SR total score of >5)
Wang et al., 2020 [28]	Residual symptoms were considered present if patient felt to have recovered by 50% or more via VAS assessment
Lambrichts et al., 2022 [25]	Decrease in MADRS score of at least 50%
Sakurai et al., 2022 [21]	Score of ≤7 on the HAMD17
Hart et al., 2023 [24]	Decrease in QIDS-SR16 composite score of ≥50% from baseline to at least one follow-up QIDS
Zhou et al., 2024 [22]	Remission was defined as a QIDS-SR16 total score of ≤5

* Definition not clearly stated, common definition delineates partial response as improvement between 25 and 50% [28]; ADT—antidepressant therapy; BDI—Beck Depression Inventory; HAM-D17—17-item Hamilton Depression Rating Scale; HANDS—Harvard Department of Psychiatry/National Depression Screening Day; HDRS-17—17-item Hamilton Depression Rating Scale; PSR—Psychiatric Rating Status; QIDS-C16—16-item Quick Inventory of Depressive Symptomatology—Clinican; QIDS-SR16—16-item Quick Inventory of Depressive Symptomatology—Self Report; VAS—Visual Analogue Scale.

## Data Availability

No new data were created or analyzed in this study. Data sharing is not applicable to this article.
